# Post Traumatic Neuroma in Continuity of the Median Nerve in a Child: A Case Report

**DOI:** 10.7759/cureus.38537

**Published:** 2023-05-04

**Authors:** Vasileios Giannatos, Sosanna Ierodiakonou, Konstantinos Koutas, Evangelia Argyropoulou, Pantelis Tsoumpos, Zinon Kokkalis

**Affiliations:** 1 Orthopedics and Traumatology, General University Hospital of Patras, Rio, GRC; 2 Orthopaedics and Traumatology, General University Hospital of Patras, Rio, GRC; 3 Orthopaedics, Karamandaneio Prefecture Children Hospital of Patras, Patras, GRC

**Keywords:** neuroma in continity, neuroma, median nerve injury, hand tumors, child

## Abstract

An 8-year-old girl suffered a wrist laceration from a sharp glass, severing the median nerve. The nerve was end-to-end repaired at the time by a pediatric orthopedic surgeon. Six months later, the girl suffered wrist pain and hyperesthesia over the previous surgical incision, significantly affecting her daily activities. Physical examination revealed a palpable mass over the median nerve with a positive Tinel sign, and the diagnosis of a painful neuroma in continuity was set. She underwent another surgery where the defective neuroma in-continuity was excised, and the median nerve was reconstructed using sural nerve cable autografts. At 18 months follow-up after the second surgery, the girl appeared with a full passive and active painless range of motion and a negative Tinel sign. This is the first case of neuroma in continuity presenting in a child in the literature, successfully managed surgically.

## Introduction

A broad spectrum of tumoral lesions may occur in the hand or wrist, from bony, soft tissue, nerve, or vascular origin [1,2]. Peripheral nerve neuromas are one of the most common peripheral nerve tumors, forming benign masses that are usually developed after trauma. After a traumatic incident, the severed nerve may continue to enlarge, forming a tumoral growth called neuroma, while the distal nerve segment undergoes Wallerian degeneration [3]. A neuroma consists of a disorganized fibro-neural tissue mass containing axons, connective tissue, and different types of cells that contract and contribute to pain [4]. Hence, the formed neuroma, usually surrounded by fibrous tissue, is painful when stimulated.

Moreover, mechanical and chemical irritation and the development of scar tissue can also contribute to neuropathic pain [5]. There are two types of neuroma lesions; one is characterized by severed nerve ends, and the other is described as neuroma in continuity. The treatment options remain challenging, especially for the neuroma in continuity [6,7].

## Case presentation

**Figure 1:**
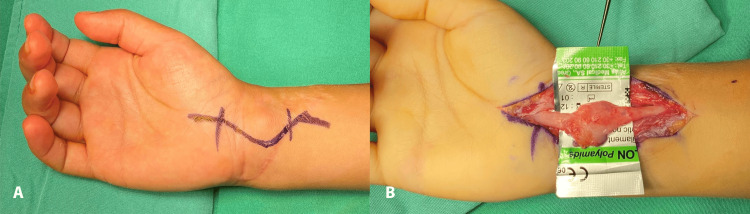
Neuroma in the continuity of the median nerve, A: Skin incision over the suspected neuroma, B: The neuroma was carefully dissected and isolated.

**Figure 2:**
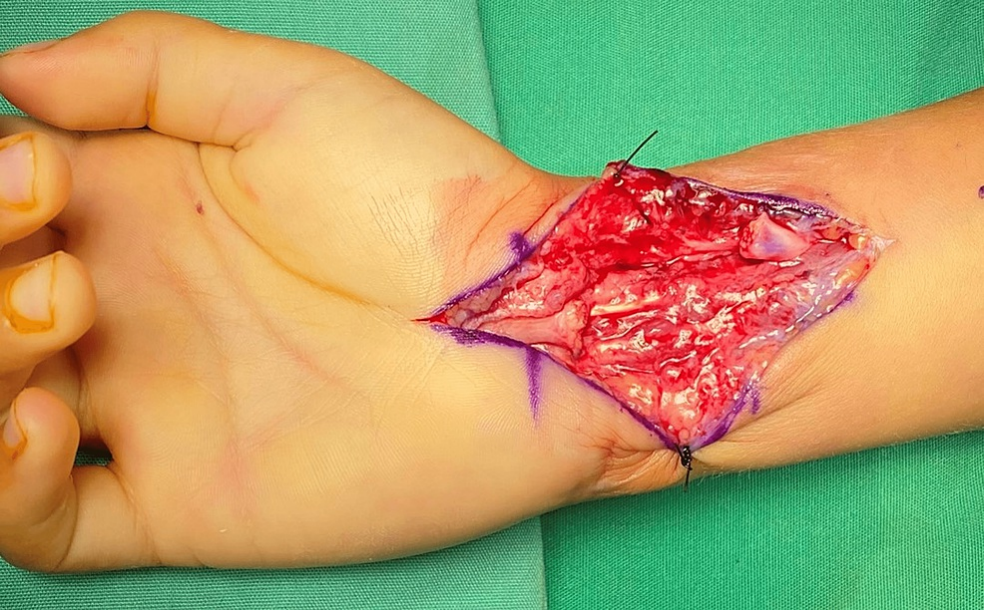
The neuroma was investigated and completely resected.

An 8-year-old girl accidentally sustained a deep laceration following sharp trauma with glass on the palmar wrist surface of her right hand in October 2020. As a result, the median nerve and the palmaris longus were completely transected. At the time of injury, the nerve was end-to-end sutured by a pediatric orthopedic surgeon, and the wrist was immobilized using a splint for a month. The splint was removed after one month, and the passive and active range of motion was increased during the following weeks. The girl reported no previous medical history. Six months after the injury, she presented to our Orthopaedic department with constant wrist pain and severe hyperesthesia over the previous surgical incision, which restricted her daily activities. The girl was very sensitive to any contact from her classmates and didn’t want to go to her previous gymnastics. After physical examination, a positive Tinel sign and a tumor on palpation were noted on the site of injury. Therefore, diagnosing a painful post-traumatic neuroma in the continuity of the median nerve was clinically established. The selected decision of treatment was surgical, seven months after the initial injury. A lazy S incision (approximately 5 cm) over the suspected neuroma was performed (Figure 1A). After careful tissue dissection, the neuroma was recognized and isolated (Figure 1B). A nerve stimulator (Vari Stim® III, nerve locator, Medtronic Xomed, Inc., Jacksonville, FL) was used to assess reserved nerve motor function across the neuroma. No action potentials were recorded, and the neuroma was completely resected at the level of healthy nerve tissue, both proximally and distally (Figure 2). A nerve gap of about 3 cm was left behind.

**Figure 3:**
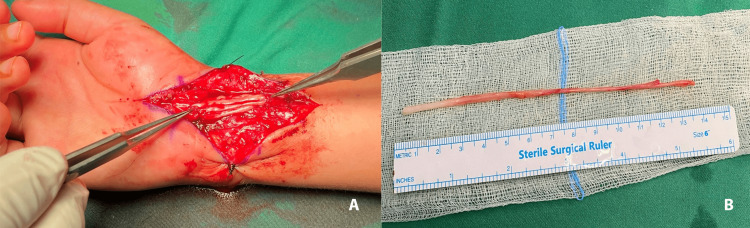
The sural autograft. A: After being utilized as cable nerve grafts, B: after being harvested.

**Figure 4:**
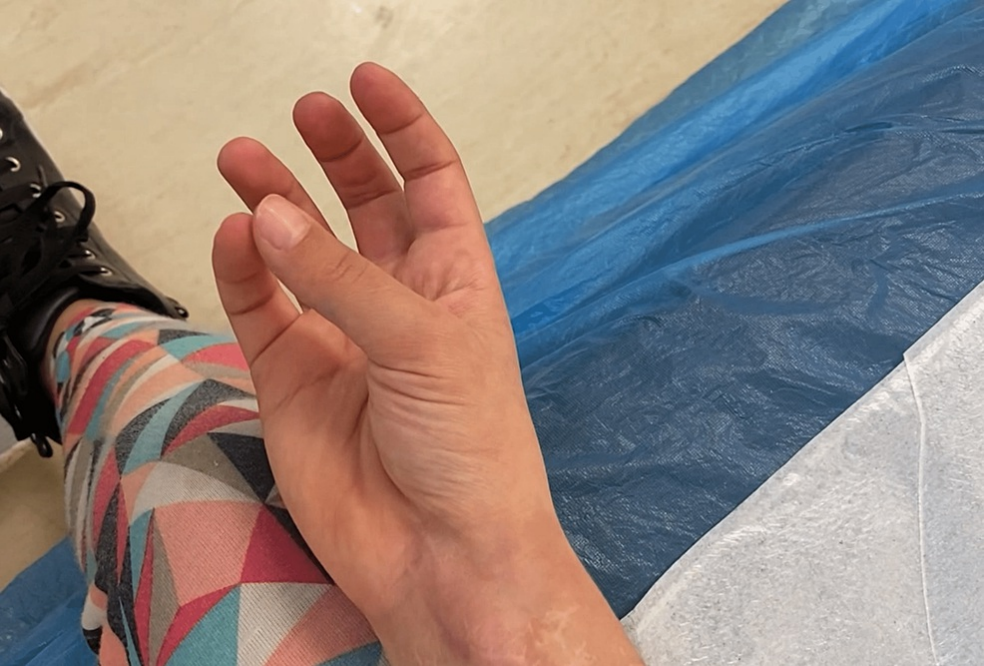
Full painless range of motion 18 months after the neuroma excision.

Thus, a sural autograft was harvested from her right distal tibia (approximately 12 cm long) and segmented into 4 parts of 3 cm each. The 4 parts of the sural autograft were utilized as cable nerve grafts to bridge the median nerve. The nerve grafts were sutured using nylon 9-0 suture without any tension (Figures 3A, 3B). At the last follow-up, 18 months after the second surgery, the girl presented with a full painless range of motion (Figure 4). Full wrist functionality and a negative Tinel sign were observed during the physical examination. The patient could return to her previous daily activities and competitive gymnastics. Verbal consent was acquired from her parents for this case presentation.

## Discussion

The diagnosis of neuromas is usually set clinically based on a history of sharp trauma, crush, or stretch injury and physical examination findings. The presence of a positive Tinel sign along with pain related to a single peripheral nerve distribution, with or without accompanying numbness or impaired sensation, may also be useful in localizing the lesion. Local anesthetic injection and imaging utilizing ultrasound or high-field MRI can also assist in increasing the specificity of the diagnosis [3,5].

Although the treatment of neuromas is mostly surgical, especially in neuromas in continuity, there is also the option of non-surgical intervention in patients with preserved distal function or adequate symptom control with conservative measures. The non-surgical approach includes pain management, physical therapies, neurorehabilitation, psychological support, and neuromodulation, such as pregabalin and gabapentin [5,8]. Should conservative treatment fail, the surgical option should be considered. Brogan et al. suggests four surgical options: (1) resection of the neuroma and possible end-to-end repair, (2) use of nerve grafts to reconnect severed proximal and distal stumps, (3) containment of the neuroma and (4) translocation of the nerve [9]. Diathermy and cryosurgery are some other treatments that are still under research. The most appropriate surgical treatment for neuromas in continuity depends on whether the distal function is preserved or not. In cases with preserved function, neurolysis is preferred. When there is limited or no distal function, resection of the damaged nerve segment and reconstruction with nerve grafts or processed nerve allografts must be performed [8]. Another interesting option supported by several studies is using a pronator quadratus flap to cover the neuroma in-continuity of the median nerve if it is located at the level of the wrist [9-11].

Terzis and Kokkalis researched to study the outcomes of secondary reconstruction of ulnar nerve lesions [12]. They compared different surgical techniques for treating ulnar nerve lesions, as well as other factors, such as the length of the graft and the patient’s age, that led to the best results. Neurolysis, secondary end-to-end suture repair, and graft repair were the methods studied, and they concluded that in case of absent sensory or motor function or loss of compound action potentials across the nerve, the use of short nerve autografts < 5cm presented the best outcomes regarding the motor and sensory function of the nerve. In cases of preserved function, however, neurolysis accompanied by other procedures shows favorable results [6-7,13]. Elliot D et al. [13] supported the use of neurolysis followed, however by wrapping the nerves in vascularized forearm fascia for the elimination of neuropathic pain, which originates from the median or ulnar nerve and is caused by direct injury, with or without repair, or scar formation because of injury to adjacent structures. Kokkalis et al. has also extensively studied the utilization of collagen nerve wrap for median nerve scarring with excellent clinical outcomes and no adverse events noted. [14, 15]. Keir Johnson et al. [7] reported a 21-year-old man employed as a mechanic with numerous congenital disorders who presented with progressive elbow pain and palpable left anterior elbow mass. Traumatic median nerve neuroma in-continuity was the diagnosis, and dissection followed by neurolysis and nerve wrapping was performed. Another case of a post-traumatic neuroma of the median nerve, which was separated from the surrounding scar tissue and then excised, was reported by Aslan et al. [6].

The treatment of neuromas in continuity in adults is thoroughly described in the literature as presented above. However, during our search, only a few papers regarding the presence of neuromas in continuity in the pediatric population were noted, mostly regarding neuromas in the brachial plexus, as described by Terzis and Kokkalis, and no papers described the presence of neuromas in continuity in peripheral nerves or the median nerve in particular. [16]. It is well established, however, that children present a higher regenerative capacity for peripheral nerve injuries [17].

## Conclusions

Neuromas of the median nerve can be treated in conservative or surgical ways, depending on the type and the preservation of the distal motor and sensory function. Neuromas in continuity present a lower threshold for surgical treatment, especially if they are associated with impaired function, sensation, or poor symptom control. In cases of preserved function, neurolysis can be performed solely. In case of sensory or motor function loss, or loss of compound action potentials across the median nerve, resection of the damaged nerve segment and reconstruction with nerve grafts are indicated. We present a case of a post-traumatic median nerve neuroma in continuity on a young girl, treated successfully with nerve dissection and reconstruction utilizing a sural cable nerve autograft.
